# A Survey of Current Practice and Perspectives on Lymphadenectomy in Minimally Invasive Surgery for Endometrial Cancer in Japan

**DOI:** 10.1111/jog.70138

**Published:** 2025-11-16

**Authors:** Masafumi Toyoshima, Satoru Kyo, Akihito Horie, Eiji Kobayashi, Yoshito Terai, Tsuyoshi Yamashita, Takuma Fujii, Hironori Asada, Yasuhisa Terao, Kentaro Sekiyama, Kenbun Sone, Masaki Mandai, the Committee for Gynecologic Oncology Surgery, Japan Society of Gynecologic and Obstetric Endoscopy and Minimally Invasive Therapy (JSGOE)

**Affiliations:** ^1^ Department of Obstetrics and Gynecology, Nippon Medical School Tokyo Japan; ^2^ Department of Obstetrics and Gynecology Shimane University Izumo Shimane Japan; ^3^ Department of Obstetrics and Gynecology Medical Research Institute KITANO HOSPITAL Osaka Japan; ^4^ Department of Obstetrics and Gynecology Oita University Oita Japan; ^5^ Department of Obstetrics and Gynecology Kobe University Graduate School of Medicine Kobe Japan; ^6^ Department of Obstetrics and Gynecology Hakodate Municipal Hospital Hakodate Hokkaido Japan; ^7^ Department of Obstetrics and Gynecology Fujita Health University Okazaki Medical Center Okazaki Aichi Japan; ^8^ Department of Obstetrics and Gynecology Shin‐Yurigaoka Hospital Kawasaki Japan; ^9^ Department of Obstetrics and Gynecology Juntendo University Tokyo Japan; ^10^ Department of Obstetrics and Gynecology KINDAI University Nara Hospital Ikoma Nara Japan; ^11^ Department of Obstetrics and Gynecology University of Tokyo Tokyo Japan; ^12^ Department of Obstetrics and Gynecology Kyoto University Kyoto Japan

**Keywords:** endometrial cancer, lymphadenectomy, minimally invasive surgery, sentinel lymph node biopsy, survey and questionnaires

## Abstract

**Objective:**

This study investigated the reasons behind the decreasing trend of lymph node dissection for endometrial cancer (EC) in Japan, focusing on the impact of minimally invasive surgery (MIS) adoption, evolving clinical guidelines, and physician work‐style reform.

**Methods:**

A cross‐sectional survey of the Japan Society of Gynecologic Oncology and Endoscopy (JSGOE) members was conducted to investigate facility demographics, MIS adoption, lymphadenectomy practices, factors influencing omission, impact of work‐style reform, and perspectives on future EC management, such as molecular classification and sentinel lymph node biopsy (SLNB).

**Results:**

In total, 424 responses were received, representing a response rate of 67.8%. MIS adoption for EC is widespread in Japan, with laparoscopy preferred over robotic surgery. Lymphadenectomy is commonly performed; however, the criteria for omission varied among institutions, with clinical guidelines published by the Japanese Society of Gynecologic Oncology having the greatest impact. Physician work‐style reform significantly affected surgical practices such as surgical scheduling, adherence to time limits, and the number of surgeons participating in surgeries, while it had little impact on the criteria for lymphadenectomy omission. The adoption of molecular classifications is increasing with approximately half of the institutions planning to implement or having partially implemented them, while SLNBs remained relatively low.

**Conclusion:**

This study highlights the significant impact of evolving clinical guidelines on lymphadenectomy practices for MIS for EC in Japan, and the limited impact of physician work‐style reform.

## 1 Introduction

Endometrial cancer (EC) is the most common gynecological malignancy in developed countries, and its incidence continues to rise globally [1, 2]. Minimally invasive surgery (MIS), including laparoscopy and robotic surgery, has emerged as the preferred surgical approach for EC. This shift is attributable to the advantages of MIS over traditional laparotomy, including reduced morbidity, shorter hospital stays, and improved cosmesis [3, 4, 5]. These benefits have led to widespread adoption of MIS for EC management.

Accurate risk assessment and treatment planning in EC necessitate complete surgical staging, a process that critically includes lymphadenectomy [6]. Lymph node dissection plays a crucial role in the accurate staging and identification of patients who may benefit from adjuvant therapy. However, the optimal extent of lymphadenectomy, particularly the necessity of pelvic and para‐aortic lymphadenectomy, remains a subject of ongoing debate. Furthermore, the safe omission of lymphadenectomy in low‐risk patients is an area of active investigation [7, 8, 9].

Recent progress in molecular profiling has led to the development of novel classification systems for EC, notably The Cancer Genome Atlas (TCGA) classification and the Proactive Molecular Risk Classifier for Endometrial Cancer (ProMisE) [10, 11]. These classification systems offer valuable prognostic insights and can guide treatment decisions, including those regarding lymphadenectomy extent or omission [12, 13]. Concurrently, sentinel lymph node biopsy (SLNB) has emerged as a less‐invasive alternative to complete lymphadenectomy for detecting lymph node metastasis in patients with EC [14, 15]. Molecular classification and SLNB are increasingly being explored as tools to refine surgical staging strategies and potentially guide the selective omission of lymphadenectomy.

Although lymphadenectomy is an essential component of EC staging, a recent database analysis of EC cases revealed a significant decrease in the number of patients undergoing lymphadenectomy in Japan [16]. This trend is concerning as lymphadenectomy omission may lead to undertreatment, potentially increasing the risk of recurrence and compromising survival. This observed decreasing trend in lymphadenectomy highlights a divergence in clinical practice that warrants investigation.

This study aimed to comprehensively examine the current practices and perspectives regarding lymphadenectomy in MIS for EC among Japanese gynecological oncologists, specifically focusing on the factors influencing the observed decreasing trend. Utilizing a nationwide cross‐sectional survey of the Japan Society of Gynecologic Oncology and Endoscopy (JSGOE) members, this study sought to determine the prevalence of MIS, describe current lymphadenectomy practices, and identify factors associated with the omission of lymphadenectomy, including the influence of evolving clinical guidelines, the introduction of MIS, and the impact of physician work‐style reform.

## 2 Materials and Methods

This study employed a cross‐sectional questionnaire survey conducted among JSGOE members. Data were collected using an online questionnaire between October 11, 2024 and November 8, 2024. The survey, which was developed in Japanese, was distributed electronically via email to eligible participants. A link to the survey form, along with an explanation of the purpose and details, was sent to all JSGOE members. The sampling frame for this study comprised all 625 JSGOE‐registered facilities. Only one response per facility was accepted. To enhance the reliability of the self‐reported data and ensure accountability, respondents were required to provide their facility's name and their name to enable potential follow‐up if needed. While the study population was limited to JSGOE member institutions, these facilities represent a significant proportion of specialized centers in Japan actively involved in gynecologic oncology and MIS where lymphadenectomy for gynecological malignancies is predominantly performed.

The survey instrument was developed by the Committee for Gynecologic Oncology Surgery of the JSGOE. The questionnaire was designed based on a review of the literature and clinical guidelines, and it underwent pilot testing with 11 gynecological oncologists to ensure clarity and comprehensiveness. The final 26‐item questionnaire encompassed the following domains: facility name, respondent name, facility size, number of specialists, current status of MIS, changes in surgical procedures for endometrial cancer (and reasons for such changes), impact of physician work‐style reform, and future prospects for endometrial cancer treatment. Specifically, survey items were designed to capture nuances in practice, such as the definition or criteria used for “partial implementation” of molecular classification. The full questionnaire is presented in Table 1, and the complete survey data are available in Table S1.

**TABLE 1:** Content of the questionnaire.

1. Facility information and respondent	
Q1.	Please enter the name of your facility.
Q2.	Please enter the name of the respondent to this questionnaire (one representative per facility).
Q3.	Please provide the number of gynecological beds in your facility.
	1–9 10–19 20–29 30–39 40–49 ≧ 50
Q4.	Please provide the number of gynecological surgeries per month at your facility.
	1–9 10–19 20–29 30–39 40–49 ≧ 50
Q5.	Please provide the number of qualified gynecological endoscopist (laparoscopy) by the JSGOE.
	0 1–2 3–4 5–6 ≧ 7
Q6.	Please provide the number of robotic surgery console surgeon certificate holders at your facility.
	0 1–2 3–4 5–6 ≧ 7
Q7.	Please provide the number of surgeons certified for robot‐assisted surgery.
	0 1–2 3–4 5–6 ≧ 7
Q8.	Please provide the number of board‐certified gynecological oncologists by the JSGO.
	0 1–2 3–4 5–6 ≧ 7
2. Current status of laparoscopic and robotic surgery for EC	
Q9.	Does your facility perform laparoscopic or robotic‐assisted surgery for endometrial cancer?
	No endometrial cancer surgery is performed. Endometrial cancer surgery is performed only by laparotomy. Endometrial cancer surgery is performed by both laparoscopy and robotic assistance. Endometrial cancer surgery is performed only by laparoscopy. Endometrial cancer surgery is performed only by robotic assistance.
Q10.	Please provide the ratio of laparoscopy to robotic assistance in endometrial cancer surgery.
	Laparoscopic surgery is more common Robotic‐assisted surgery is more common They are almost equal
Q11.	If there are no contraindications for MIS, please select the main surgical procedure for each of the following endometrial cancer cases categorized by open surgery, laparoscopic surgery, and robotic surgery: Endometrioid carcinoma G1/G2, stage Ia, no myometrial invasion
	Total hysterectomy + bilateral adnexectomy Total hysterectomy + bilateral adnexectomy + pelvic lymph node dissection Total hysterectomy + bilateral adnexectomy + pelvic lymph node dissection + para‐aortic lymph node dissection No surgery
Q12.	If there are no contraindications for MIS, please select the main surgical procedure for each of the following endometrial cancer cases categorized by open surgery, laparoscopic surgery, and robotic surgery: Endometrioid carcinoma G1/G2, stage Ia, with myometrial invasion
	Total hysterectomy + bilateral adnexectomy Total hysterectomy + bilateral adnexectomy + pelvic lymph node dissection Total hysterectomy + bilateral adnexectomy + pelvic lymph node dissection + para‐aortic lymph node dissection No surgery
Q13.	If there are no contraindications for MIS, please select the main surgical procedure for each of the following endometrial cancer cases categorized by open surgery, laparoscopic surgery, and robotic surgery: Special histological type, endometrioid carcinoma G3, stage Ia, no myometrial invasion
	Total hysterectomy + bilateral adnexectomy Total hysterectomy + bilateral adnexectomy + pelvic lymph node dissection Total hysterectomy + bilateral adnexectomy + pelvic lymph node dissection + para‐aortic lymph node dissection Total hysterectomy + bilateral adnexectomy + pelvic lymph node dissection + para‐aortic lymph node dissection + omentectomy No surgery
Q14.	If there are no contraindications for MIS, please select the main surgical procedure for each of the following endometrial cancer cases categorized by open surgery, laparoscopic surgery, and robotic surgery: Special histological type, endometrioid carcinoma G3, stage Ia, with myometrial invasion
	Total hysterectomy + bilateral adnexectomy Total hysterectomy + bilateral adnexectomy + pelvic lymph node dissection Total hysterectomy + bilateral adnexectomy + pelvic lymph node dissection + para‐aortic lymph node dissection Total hysterectomy + bilateral adnexectomy + pelvic lymph node dissection + para‐aortic lymph node dissection + omentectomy No surgery
Q15.	Please select the choice of additional treatment if surgery with lymph node dissection omitted results in undertreatment (e.g., unexpectedly deep myometrial invasion or cervical gland involvement).
	Basically, additional surgery is performed No surgery is performed; however, chemotherapy is administered Individual response Other
3. Changes in surgical procedures and reasons	
Q16.	After the introduction of laparoscopic or robotic‐assisted surgery for endometrial cancer, have there been any changes in surgical procedures compared to laparotomy?
	Yes No Cannot say either way
Q17.	Please select the reason for the change in surgical procedure from the following (multiple selections possible).
	Description in the endometrial cancer treatment guidelines or the gynecological endoscopic surgery guidelines Technical problems with laparoscopic or robotic‐assisted surgery
	Insurance coverage Because it takes time to operate Personnel problems (including changes in the number of working physicians and the introduction of work style reforms for physicians) Other
Q18.	Please provide the specific changes in surgical procedures (multiple selections possible).
	The indications for lymph node dissection have changed The method of hysterectomy has changed The technique of lymph node dissection has changed The indications for surgery have changed Other
Q19.	Please provide the criteria for omitting pelvic lymph node dissection at your institution in laparoscopic or robotic‐assisted endometrial cancer surgery (multiple selections possible).
	Clinical stage Histological type of endometrial cancer Severe obesity Complications of other organs Elderly Pelvic lymph node dissection is never omitted Other
Q20.	Please provide the criteria for omitting para‐aortic lymph node dissection at your institution in laparoscopic or robotic‐assisted endometrial cancer surgery (multiple selections possible).
	Clinical stage Histological type of endometrial cancer Severe obesity Complications of other organs Elderly Para‐aortic lymph node dissection is never omitted Other
4. Impact of physician work‐style reform	
Q21.	Has the “physician work‐style reform” introduced in April 2024 had any impact on gynecological surgery at your facility?
	Yes No Cannot say either way
Q22.	Please specifically describe the impact of “physician work‐style reform” on gynecological surgery (multiple selections possible).
	Affecting the scheduling of surgeries Changed surgical indications and procedures Changed lymph node dissection criteria
	Stricter regulations on surgery completion time Changed the number of people participating in surgery Other
5. Future Plans	
Q23.	Does your facility use The Human Cancer Genome Atlas (TCGA)‐based molecular genetic classification or ProMiSE classification for endometrial cancer?
	In use Partially in use (e.g., p53 immunostaining) Not currently in use, but planned for the future No plans to introduce at this time
Q24.	Technetium (99mTc) Tilmanocept has been listed in the National Health Insurance Reimbursement Schedule as a tracer for sentinel lymph nodes in endometrial cancer surgery, but sentinel lymph node biopsy surcharges, which are procedure fees, are not recognized (as of September 2024). Does your facility currently perform “sentinel lymph node biopsy” in endometrial cancer surgery?
	Yes No
Q25.	If the sentinel lymph node biopsy is negative, do you omit lymph node dissection?
	Yes No (backup lymph node dissection is performed)
Q26.	Would you like to introduce sentinel lymph node biopsy in endometrial cancer surgery at your facility in the future?
	I think I will I don't think I will I can't say either way

Descriptive statistics were used to summarize the survey responses. Categorical variables were presented as frequencies and percentages. Exploratory subgroup analyses, such as stratified analysis by facility size or presence of certified MIS surgeons/gynecological oncologists, were also conducted to provide further context to the findings where appropriate.

This study was conducted according to the ethical principles of the Declaration of Helsinki. The study was approved by the Board of Directors of the JSGOE before its commencement. The study protocol was approved by the Institutional Review Board of Nippon Medical School Hospital (B‐2023‐647). Given the nature of this survey research, utilizing anonymized questionnaire data without patient‐specific information or intervention, the requirement for individual informed consent was waived by the approving Institutional Review Board.

## 3 Results

**FIGURE 1:**
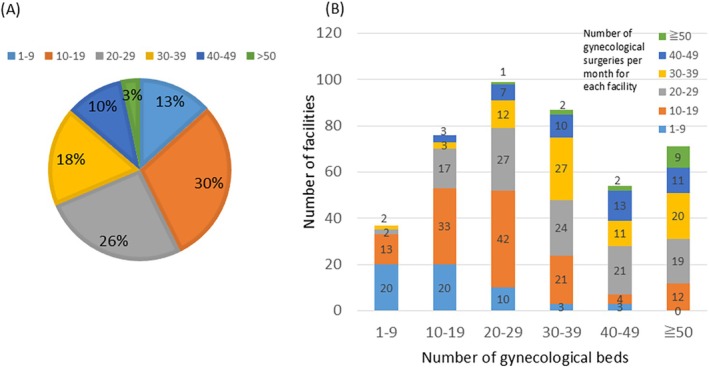
Characteristics of facilities: Gynecological surgery volume and bed capacity. (A) Percentage distribution of monthly gynecological surgeries per facility. (B) Number of facilities by monthly gynecological surgery volume, stratified by the number of gynecological beds.

In total, 424 responses were received. This represented a high response rate of 67.8%. Regarding the number of monthly gynecological surgeries, 10–19 cases were the most frequent (30%), followed by 20–29 cases (26%) (Figure 1A). High‐volume facilities with 50 or more cases accounted for 3%. For the number of gynecological beds at each facility, 20–29 beds were the most common, followed by 30–39 beds, and then 50 or more beds (Figure 1B).

To assess the availability of specialized expertise at each facility, the number of qualified professionals, including Japan Society of Gynecologic Oncology (JSGO)‐certified gynecological oncologists, JSGOE‐certified laparoscopic surgeons, and robot‐assisted surgeons, was surveyed. The distribution of JSGO‐certified gynecological oncologists revealed that 29.7% of the facilities had no certified oncologists (Table 2). The distribution of JSGOE‐certified laparoscopic surgeons revealed that most facilities (55.7%) had 1–2 certified surgeons, whereas 13.9% had none. In contrast, the distribution of certified robotic surgeons showed that a substantial proportion of facilities (65.8%) had no certified robotic surgeons, with 28.8% having 1–2 certified surgeons.

**TABLE 2:** Number of certified surgeons per facility (*N* = 424).

	0	1–2	3–4	5–6	≥ 7
Board‐certified gynecologic oncologist by the JSGO at each facility	126 (29.7%)	194 (45.8%)	62 (14.6%)	23 (5.4%)	19 (4.5%)
Qualified gynecologic endoscopist (laparoscopy) by the JSGOE	59 (13.9%)	236 (55.7%)	89 (21.0%)	25 (5.9%)	15 (3.5%)
Surgeons certified robot‐assisted surgery	279 (65.8%)	122 (28.8%)	17 (4.0%)	5 (1%)	1 (0.2%)

Data on surgical approach was analyzed based on the presence of certified gynecological oncologists and MIS surgeons. In this cohort, 126 facilities lacked gynecological oncologists, 59 lacked certified MIS surgeons, and 32 lacked both (Table 3). The choice of surgical procedure for early‐stage EC differed between all facilities and those without gynecological oncologists. Overall, 23.3% of facilities did not perform surgery for endometrial cancer, 17.9% performed only open surgery, and the majority (total 58.7%) performed surgery using MIS. In facilities without a JSGO‐certified gynecological oncologist, 29.7% of facilities did not perform endometrial cancer surgery, 45.8% performed only open surgery (the most frequent), and a total of 20% performed surgery using MIS. In facilities without a certified MIS surgeon, the highest proportion (40.7%) did not perform endometrial cancer surgery. In facilities without both a JSGO‐certified gynecological oncologist and a certified MIS surgeon, the highest proportion (59.4%) did not perform endometrial cancer surgery. MIS for EC was rarely performed in this group.

**TABLE 3:** Percentage of surgical options for early‐stage endometrial cancer in all facilities and in Facilities without gynecologic oncologists.

	We do not perform surgery for endometrial cancer	We only perform open surgery for endometrial cancer	We perform endometrial cancer surgery both laparoscopically and with robotic assistance	We perform endometrial cancer surgery laparoscopically only	We perform endometrial cancer surgery with robotic assistance only	Total
All facilities	99 (23.3%)	76 (17.9%)	165 (38.9%)	80 (18.9%)	4 (0.9%)	424
(1) No board‐certified gynecologic oncologist by the JSGO	62 (29.7%)	26 (45.8%)	22 (14.6%)	1 (5.4%)	0 (0%)	126
(2) No qualified gynecologic endoscopist (laparoscopy) by the JSGOE nor Surgeons certified robot‐assisted surgery	24 (40.7%)	23 (39.0%)	7 (11.9%)	2 (3.4%)	1 (1.7%)	59
(1) and (2)	19 (59.4%)	12 (37.5%)	0 (0%)	1 (3.1%)	0 (0%)	32

**FIGURE 2:**
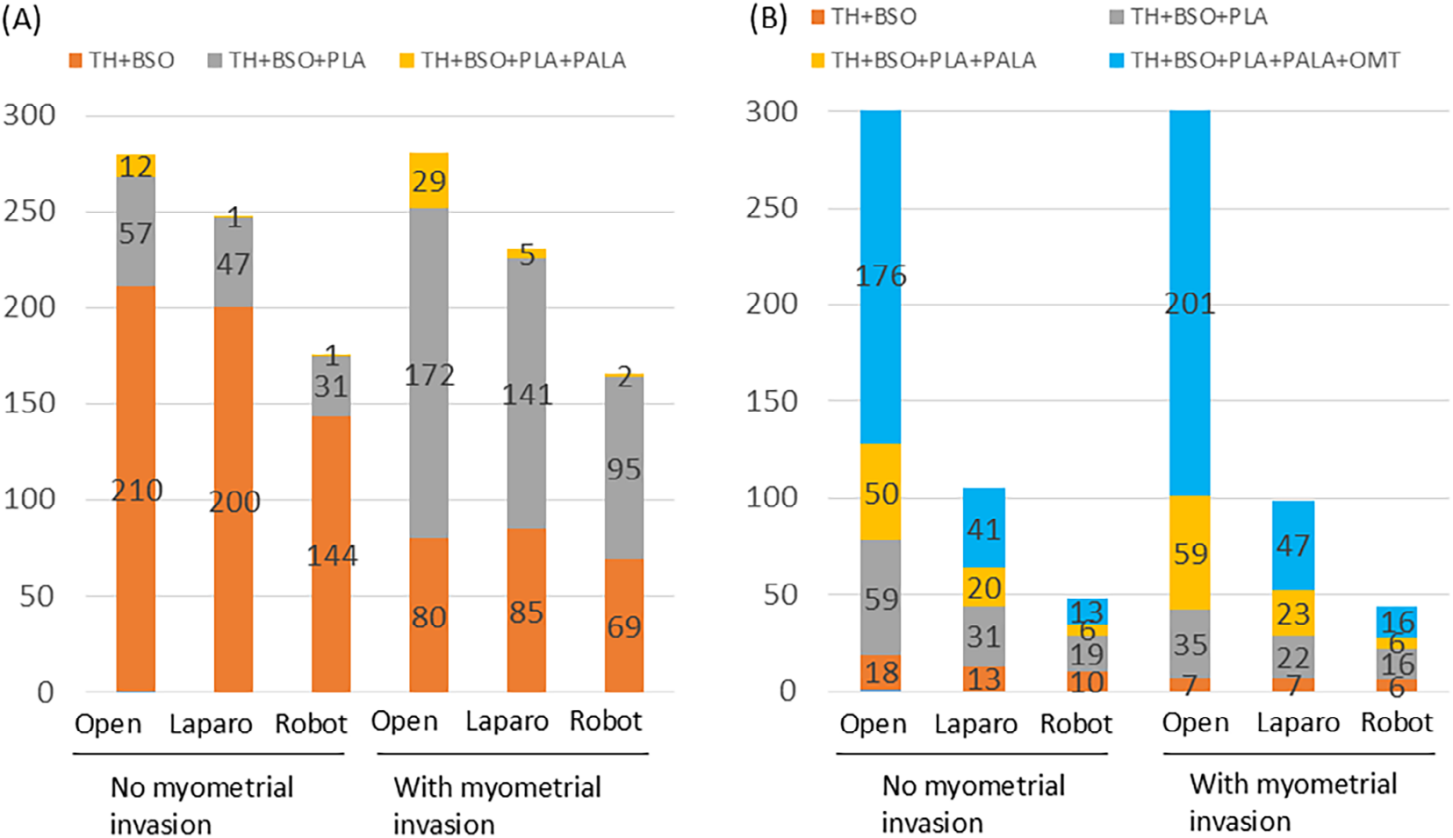
Differences in lymph node dissection practices based on the surgical procedure and the presence or absence of myometrial invasion in Stage IA endometrioid carcinoma. (A) Lymph node dissection practices for Endometrioid carcinoma G1/G2, FIGO Stage IA patients. (B) Lymph node dissection practices for Endometrioid carcinoma G3, specific histological types, FIGO Stage IA patients. ALA, para‐aortic lymphadenectomy; BSO, Bilateral salpingo‐oophorectomy; FIGO, The International Federation of Gynecology and Obstetrics; OMT, omentectomy; PLA, pelvic lymphadenectomy; TAH, total hysterectomy.

We analyzed surgical procedures performed (open, laparoscopic, and robotic) for early‐stage EC, stratified by specific stage and histological type. For the International Federation of Gynecology and Obstetrics (FIGO) Stage IA endometrioid carcinoma Grade 1 (G1)/G2 cases, a tendency for lymph node dissection omission was observed across all surgical approaches when myometrial invasion was not expected (Figure 2A). Although MIS was widely performed for these cases, laparoscopic surgery was more common than robotic surgery. When myometrial invasion was expected, lymphadenectomy was widely performed regardless of the surgical approach; however, a higher proportion of facilities omitted lymphadenectomy with MIS compared to open surgery. For FIGO Stage IA cases of endometrioid carcinoma G3 or specific histological types, open surgery was frequently performed regardless of the presence or absence of myometrial invasion (Figure 2B). In this group, surgical procedures including lymph node dissection and omentectomy were most widely performed, and omission of lymph node dissection was uncommon.

**FIGURE 3:**
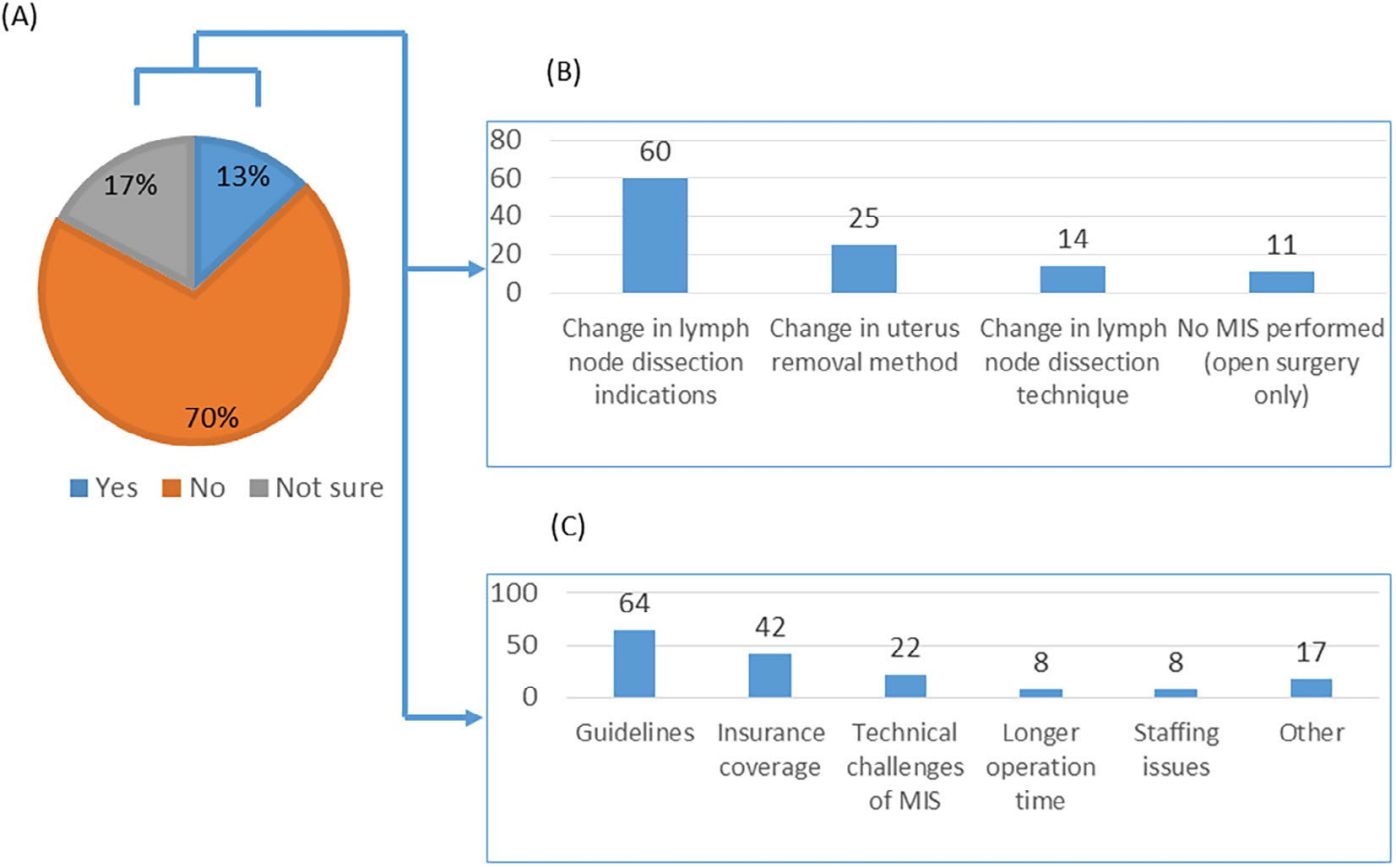
Impact of MIS on surgical approaches for endometrial cancer. (A) Proportion of facilities reporting changes in surgical procedures after MIS introduction. (B) Specific types of changes reported in surgical procedures. (C) Reasons cited for changes in surgical procedures.

The influence of MIS introduction on surgical procedures for EC was investigated. Although most respondents (70%) reported no changes, a notable proportion (13%) indicated changes (Figure 3A), primarily in the indications for lymphadenectomy and the approach to hysterectomy (Figure 3B). The most common reasons cited for changes in surgical procedures were descriptions in various guidelines and insurance coverage in Japan (Figure 3C).

**FIGURE 4:**
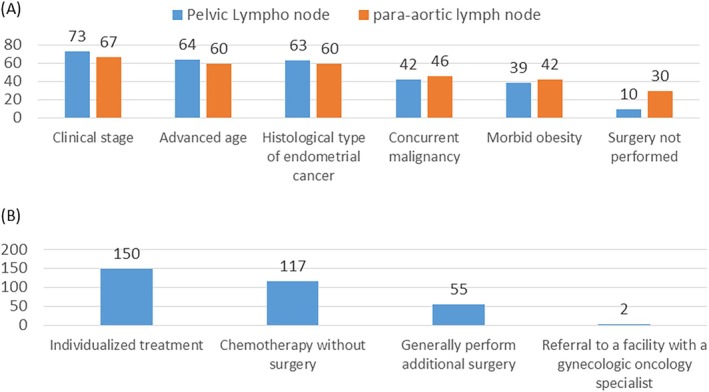
Lymph node dissection omission criteria in MIS for endometrial cancer. (A) Reported criteria for omitting lymph node dissection in MIS for endometrial cancer. (B) Preferred treatment options for potential undertreatment after omitted lymph node dissection.

The criteria for omitting lymphadenectomy in MIS for EC varied among the respondents, with clinical stage, advanced age, histological type, concurrent malignancy, and morbid obesity being the most common factors (Figure 4A). In cases of undertreatment due to omitted lymphadenectomy, most facilities preferred individualized assessment, with additional chemotherapy favored over additional surgery (Figure 4B).

**FIGURE 5:**
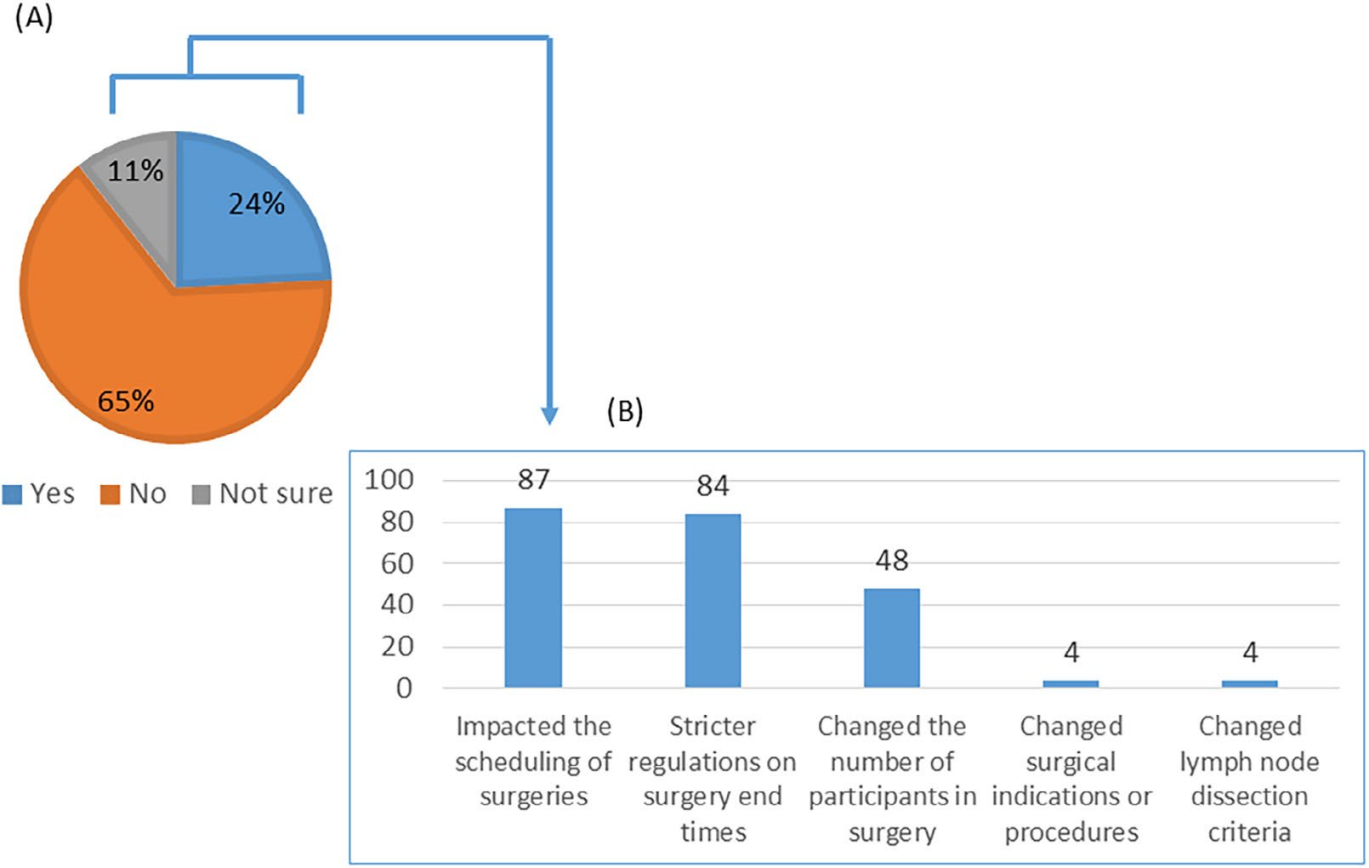
Impact of the “physician work‐style reform” on gynecological surgery. (A) Proportion of facilities reporting impact of physician work‐style reform on gynecological surgery. (B) Specific types of impact reported from physician work‐style reform.

The effects of the “work‐style reform for physicians” on gynecological surgery were assessed. Although most respondents (65%) reported no impact, a significant proportion (24%) experienced changes (Figure 5A), primarily in surgical scheduling, adherence to time limits, and the number of surgeons participating in surgeries (Figure 5B).

Furthermore, future perspectives regarding the adoption of molecular pathological classification and SLNBs for EC were explored. Although several facilities have not yet implemented molecular classification, a substantial proportion plan to introduce it in the future or have already partially implemented it (Table 4). In terms of SLNBs, most facilities expressed a desire to introduce it in the future (63/137), although a significant proportion (23/137) had no plans to do so (Table 4).

**TABLE 4:** Implementation of SLNB in endometrial cancer diagnosis.

Question	Answer choices	1–9	10–19	20–29	30–39	40–49	≥ 50	Total
Has your institution implemented molecular genetic classification using TCGA or ProMisE classification for endometrial cancer? (*n* = 147)	Not currently planned for implementation	4 (5.1%)	12 (15.4%)	23 (29.5%)	17 (21.8%)	11 (14.1%)	11 (14.1%)	78
	Not currently implemented, but planned for future implementation	1 (2.8%)	3 (8.3%)	7 (19.4%)	8 (22.2%)	8 (22.2%)	9 (25%)	36
	Partially implemented	0 (0%)	3 (10.3%)	2 (6.9%)	9 (31.0%)	9 (31.0%)	6 (20.7%)	29
	Implemented	1 (25%)	1 (25%)	0 (0%)	0 (0%)	0 (0%)	2 (50%)	4
Do you plan to introduce sentinel lymph node biopsy for endometrial cancer surgery at your facility in the future? (*n* = 137)	Yes	1 (1.6%)	8 (12.7%)	12 (19.0%)	12 (19.0%)	16 (25.4%)	14 (22.2%)	63
	No	3 (13.0%)	2 (8.7%)	6 (26.1%)	8 (34.8%)	3 (13.0%)	1 (4.3%)	23
	Not sure	1 (2.0%)	9 (17.6%)	14 (27.5%)	9 (17.6%)	9 (17.6%)	9 (17.6%)	51

## 4 Discussion

This study confirms a decreasing proportion of lymph node dissection during surgery for EC in Japan, which is consistent with previous reports [16]. This trend appears to be primarily driven by evolving clinical guidelines for EC treatment. A closer look at the JSGO guidelines for endometrial cancer treatment revealed a shift in the recommendations for lymphadenectomy. Although the 2009 version did not mention omitting lymphadenectomy [17], the 2013 version suggested the possibility of omitting this procedure in patients with early‐stage cancers [18]. The 2018 and subsequent versions explicitly recommend omitting lymphadenectomy in the low‐risk group to prevent postoperative recurrence [19, 20]. This evolution in guidelines reflects the growing body of evidence suggesting that lymphadenectomy can safely be omitted in select low‐risk patients, as demonstrated in several clinical trials.

While the proportion of MIS for EC has been increasing, our study found that the frequency of lymph node dissection differed depending on the surgical approach (open surgery vs. MIS) and the size of the medical facility [17, 21]. Several factors may contribute to the decreasing proportion of lymph node dissections in EC surgery, including the implementation of SLNBs (discussed below) and the incorporation of clinical trial results into the guidelines [22, 23]. Indeed, our results revealed that the adoption of MIS for EC is widespread in Japan. This finding is consistent with global trends and reflects the established benefits of MIS, such as reduced morbidity and improved quality of life [3, 4]. However, the choice between laparoscopy and robotic surgery may be influenced by several factors, such as surgeon experience, institutional resources, and patient characteristics. Further research is necessary to elucidate the optimal surgical approach for different EC subtypes and patient populations.

Regarding lymphadenectomy, our study revealed that although commonly performed, the criteria for omitting this procedure varied widely among respondents (Figure 4). This highlights the ongoing debate regarding the extent of lymphadenectomy and the potential for omitting this procedure in low‐risk patients. Although some studies have suggested that omitting lymphadenectomy in select patients does not compromise survival [7, 8], others have emphasized the importance of complete lymphadenectomy for accurate staging and prognostication [6]. The decision to omit lymphadenectomy should be individualized based on a comprehensive assessment of the patient and tumor factors. Consistent with the variation in criteria, patient‐related factors, such as clinical stage, age, and comorbidities were the most commonly cited reasons for omitting lymphadenectomy (Figure 4). This suggests that clinicians exercise caution when selecting patients for lymphadenectomy omission. However, the long‐term consequences of the omission of lymphadenectomy in these patients remain unclear. Future studies with longer follow‐up periods are necessary to assess the oncological safety of this approach.

Furthermore, the introduction of molecular classification systems, such as TCGA and ProMisE, may provide additional guidance in identifying patients for whom lymphadenectomy can be safely omitted [12, 13]. These classifications offer valuable prognostic information and may help stratify patients into different risk groups, allowing for more personalized treatment decisions. Molecular classification systems are increasingly being used to guide treatment decision‐making in EC. Consistent with this trend, our study found that a significant proportion of respondents plan to adopt TCGA or ProMisE in their clinical practice (Table 4). This reflects the growing recognition of the importance of molecular profiling in personalizing cancer treatment. Future studies should focus on developing evidence‐based guidelines for the use of molecular classification systems to inform surgical approaches and lymphadenectomy decisions.

The “physician work‐style reform” implemented in Japan in 2024 aimed to improve the work–life balance for physicians [24]. Our study found that this reform has had varied effects on surgical practice, with some respondents reporting a reduction in surgical volume or changes in surgical procedures, while others noted no significant impact (Figure 5). The long‐term effects of the “physician work‐style reform” on surgical training and patient care warrant further investigation.

SLNB has emerged as a less‐invasive alternative to complete lymphadenectomy for EC staging. Our study found that the adoption of SLNBs in Japan remains relatively low, with several barriers to its widespread implementation. These include the lack of reimbursement for SLNBs, concerns regarding accuracy and false‐negative rates, and the need for specialized equipment and training [14, 15]. Notably, the greatest barrier is the lack of insurance coverage in Japan. Overcoming these challenges is crucial for promoting the adoption of SLNBs and improving the quality of care for patients with EC.

This study has several limitations. First, the data were collected using a self‐reported survey, which may be subject to recall and social desirability biases. Second, while the response rate was high (67.8%), the study was conducted among JSGOE members, who may not represent all gynecological oncologists in Japan. However, these facilities represent a significant proportion of specialized centers in Japan actively involved in gynecologic oncology and MIS where lymphadenectomy for gynecological malignancies is predominantly performed. Third, this study did not collect data on patient outcomes; therefore, we could not assess the effects of different surgical approaches on patient survival or quality of life. Future studies using objective measures of surgical practice and patient outcomes should address these limitations.

In conclusion, this study provides a comprehensive overview of the current practice and perspectives on lymphadenectomy in MIS for EC in Japan. Our findings highlight that the decreasing trend in lymph node dissection is primarily driven by evolving clinical guidelines, with MIS and physician work‐style reform having limited impact. The study also emphasizes the need for individualized treatment decisions based on a thorough assessment of patient and tumor factors, highlights the importance of ongoing research to optimize surgical and adjuvant therapies, and suggests the potential of molecular classification systems and SLNB to improve the quality of care for patients with EC.

## Author Contributions

Conception and design: Masafumi Toyoshima and Satoru Kyo. Acquisition of data: Masafumi Toyoshima, Satoru Kyo, and Akihito Horie. Analysis and interpretation: Masafumi Toyoshima, Satoru Kyo, Akihito Horie, Eiji Kobayashi, Terai Yoshito, Tsuyoshi Yamashita, Takuma Fujii, Hironori Asada, Yasuhisa Terao, Kentaro Sekiyama, and Kenbun Sone. Drafting of the manuscript: Masafumi Toyoshima. Critical revision of the manuscript for important intellectual content: Satoru Kyo. Obtaining funding: Satoru Kyo and Masaki Mandai. Supervision: Satoru Kyo and Masaki Mandai. All authors have read and agreed to the published version of the manuscript.

## Conflicts of Interest

Dr. Yasuhisa Terao is an Editorial Board member of JOGR Journal and a co‐author of this article. To minimize bias, he was excluded from all editorial decision‐making related to the acceptance of this article for publication. The all other authors declare no conflicts of interest.

## Supporting information


**Table S1:** Detailed responses to the cross‐sectional survey on current practice and perspectives on lymphadenectomy in minimally invasive surgery for endometrial cancer in Japan.

## Data Availability

The data supporting the findings of this study are available within the article and its Supporting Information. The complete survey dataset is provided as Table S1.
